# Urban PM2.5 Induces Cellular Toxicity, Hormone Dysregulation, Oxidative Damage, Inflammation, and Mitochondrial Interference in the HRT8 Trophoblast Cell Line

**DOI:** 10.3389/fendo.2020.00075

**Published:** 2020-03-12

**Authors:** Åsa Nääv, Lena Erlandsson, Christina Isaxon, Eleonor Åsander Frostner, Johannes Ehinger, Moa K. Sporre, Annette M. Krais, Bo Strandberg, Thomas Lundh, Eskil Elmér, Ebba Malmqvist, Stefan R. Hansson

**Affiliations:** ^1^Obstetrics and Gynecology, Department of Clinical Sciences Lund, Lund University, Lund, Sweden; ^2^Department of Ergonomics and Aerosol Technology, Lund University, Lund, Sweden; ^3^Mitochondrial Medicine, Department of Clinical Sciences Lund, Lund University, Lund, Sweden; ^4^Department of Physics, Lund University, Lund, Sweden; ^5^Division of Occupational and Environmental Medicine, Institution of Laboratory Medicine, Lund University, Lund, Sweden

**Keywords:** placenta, trophoblast cells, air pollution, PM2.5, preeclampsia, inflammation, polycyclic aromatic hydrocarbons, mitochondria

## Abstract

**Objective:** Epidemiological studies have found air pollution to be a driver of adverse pregnancy outcomes, including gestational diabetes, low term birth weight and preeclampsia. It is unknown what biological mechanisms are involved in this process. A first trimester trophoblast cell line (HTR-8/SVneo) was exposed to various concentrations of PM2.5 (PM2.5) in order to elucidate the effect of urban particulate matter (PM) of size <2.5 μm on placental function.

**Methods:** PM2.5 were collected at a site representative of urban traffic and dispersed in cell media by indirect and direct sonication. The HTR-8 cells were grown under standard conditions. Cellular uptake was studied after 24 and 48 h of exposure by transmission electron microscopy (TEM). The secretion of human chorionic gonadotropin (hCG), progesterone, and Interleukin-6 (IL-6) was measured by ELISA. Changes in membrane integrity and H_2_O_2_ production were analyzed using the CellTox^TM^ Green Cytotoxicity and ROSGlo^TM^ assays. Protease activity was evaluated by MitoTox^TM^ assay. Mitochondrial function was assessed through high resolution respirometry in an Oroboros O2k-FluoRespirometer, and mitochondrial content was quantified by citrate synthase activity.

**Results:** TEM analysis depicted PM2.5 cellular uptake and localization of the PM2.5 to the mitochondria after 24 h. The cells showed aggregated cytoskeleton and generalized necrotic appearance, such as chromatin condensation, organelle swelling and signs of lost membrane integrity. The mitochondria displayed vacuolization and disruption of cristae morphology. At 48 h exposure, a significant drop in hCG secretion and a significant increase in progesterone secretion and IL-6 production occurred. At 48 h exposure, a five-fold increase in protease activity and a significant alteration of H_2_O_2_ production was observed. The HTR-8 cells exhibited evidence of increased cytotoxicity with increasing exposure time and dose of PM2.5. No significant difference in mitochondrial respiration or mitochondrial mass could be demonstrated.

**Conclusion:** Following exposure to air pollution, intracellular accumulation of PM may contribute to the placental dysfunction associated with pregnancy outcomes, such as preeclampsia and intrauterine growth restriction, through their direct and indirect effects on trophoblast protein secretion, hormone regulation, inflammatory response, and mitochondrial interference.

## Introduction

Particulate matter (PM) is a complex mixture of suspended primary particles and agglomerates/aggregates of e.g., transition metal oxides, ammonium nitrate, sulfates, protein complexes, and organic materials including polycyclic aromatic hydrocarbons (PAHs) (1–5). PM's toxicity is multifactorial and depends on a variety of particle properties, such as composition, size, and shape as well as number, mass and surface area concentration (1–5). PM with a diameter of <2.5 μm (PM2.5) is generally associated with combustion-related emission sources (3). This fine PM, especially the size fractions <200 nm, have a high likelihood of depositing in the lungs' alveolar region and, subsequently, entering the blood circulation through the alveoli walls via translocation (6–11).

To date, a staggering 87% of the world population resides in areas that exceed the WHO air quality guidelines for PM2.5 (12). In fact, air pollution is the single largest environmental cause of disease (13). Recent studies point toward an association between air pollution exposure and a wide range of diseases such as cardiovascular events in adults (14), cerebrovascular disease (15), and adverse pregnancy outcomes such as preeclampsia (PE) (16). Air pollution has also been associated with hypertensive disorders during pregnancy (17). Furthermore, a large number of epidemiological studies have shown an association between air pollution exposure during pregnancy and the risk of developing PE, even in areas with levels below the WHO air quality guidelines (16, 18–21).

Preeclampsia is a serious pregnancy-related multisystem syndrome, commonly defined as hypertension and proteinuria after the 20th gestational week (22). It complicates roughly 3–7% of pregnancies worldwide (23, 24) and is the main cause of maternal morbidity and mortality. PE is associated with intrauterine growth restriction (IUGR), preterm birth, and perinatal deaths. Both PE and IUGR are associated with an increased long-term risk of developing cardiovascular disease and metabolic disease later in life (25, 26). The association between air pollution exposure and PE is stipulated to be driven by the oxidative stress and systemic inflammation caused or aggravated by air pollution exposure, eventually leading to the vascular endothelial injury, which is a corner stone in the etiology of PE (14, 24, 27, 28).

Despite the wealth of epidemiological evidence associating the effects of air pollution on pregnant women and the developing fetus, air pollution is still not included in the WHO Burden of Disease studies (29). The biological mechanisms and pathways by which air pollution exposure during or prior to pregnancy contributes to the development of PE remain largely unexplored. Therefore, studies deciphering the biological mechanisms contributing to the toxicity of air pollution during pregnancy are urgently needed. By investigating the influence of fine particular matter on placental trophoblast cells, we aim to identify a possible pathway by which air pollution mediates its adverse effects on the health of pregnant women and the developing fetus.

## Materials and Methods

### Ambient PM2.5 Preparation

Ambient PM2.5 particles were collected at a central location in Malmö, southern Sweden, over 26 days in April–May 2017. This was conducted at a height of 3, 4 m from a street crossing with an average daily traffic (2017) of 28,000 vehicles. A high-volume cascade impactor (BGI900, Mesa Labs, USA) was utilized for this process. The impactor samples air (0.9 m^3^/min) and collects all particles smaller than 2.5 μm on a polypropylene filter. Subsequent particle extraction followed Mesa Labs' protocol using pure methanol, and particles were then dried in a vacuum evaporator (SpeedVac HT-4X Evaporator, GeneVac, UK).

### Characterization of PM2.5

During the sampling period, time-resolved mass concentration measurements of PM2.5 were conducted with a tapered element oscillating microbalance (TEOM 1400AB) with a filter dynamics measurement system, and of black carbon (soot) and organic carbon using light absorption with an aethalometer (AE33). The dried collected particles were analyzed by gas chromatography-mass spectrometry (GC-MS) for the presence of PAH (30). Further, the PM2.5 fraction was analyzed for metal compositions including aluminum (Al), arsenic (As), barium (Ba), cadmium (Cd), chromium (Cr), cobalt (Co), copper (Cu), iron (Fe), lead (Pb), manganese (Mn), nickel (Ni), thallium (Tl), vanadium (V), and zinc (Zn). For this, ~10 mg sample was dissolved in 1 ml concentrated nitric acid at 70°C for 16 h. After dilution with Milli-Q water, the metal concentrations were determined by inductively coupled plasma-mass spectrometry (ICP-MS; iCAP Q, Thermo Scientific, Bremen, GmbH) in collision cell mode with kinetic energy discrimination using helium as the collision gas. The detection limits, calculated as three times the standard deviation (SD) of the blank, were 0.05 ng (Mn, Ni, As, Cd, Tl, Pb), 0.06 ng (Ba), 0.07 ng (Cr, Cu,), 0.08 ng (V), 0.09 ng (Co), 0.15 ng (Zn), 0.70 ng (Fe), and 2.7 ng (Al). Additionally, wind trajectories were calculated by Hybrid Single Particle Lagrangian Integrated Trajectory (Hysplit, Air Resources Lab) model (31, 32) to investigate the local and the in-transported contribution to the collected PM2.5. Hysplit is run with gridded meteorological data from the Center of Environmental Predictions (NCEP) Global Data Assimilation System (GDAS). Local meteorological data from a monitoring station at Heleneholm, Malmö was also used to investigate the wind speeds and wind direction in Malmö situated around 1 km from the particle collection site.

### PM2.5 Dispersion

Prior to exposure experiments, 0.9 mg of dried and extracted PM2.5, as described above, was dissolved in 900 μl of cell media (CM; supplemented RPMI-1640 medium, see below) in 1.5 ml Eppendorf tubes and subjected to indirect and direct sonication. Indirect ultra sonication was performed at 4°C and at 120 W for 15 min using an Ultrasonic Cleaner water bath (Mettler Electronics), followed by direct sonication at room temperature (RT) at 50 W, 0.05 cycle, 20% amplitude for 60 s using an UP50H Ultrasonic Processor (Hielscher Ultrasound Technology). The immersion was aliquoted and diluted to desired concentration. The CM without PM, used on control cells, underwent the same protocol and maintained the same volumes. The direct sonication step followed by vortex was performed prior to each exposure.

### Cell Culture

The commercially available HTR-8 (HTR-8/SVneo) cells, a human first trimester transformed trophoblast cell line (the American Type Culture Collection, ATCC Cell Lines, CRL-3271, lot number 64275781) were maintained in HyClone RPMI-1640 medium (Fisher Scientific) supplemented with 5% fetal bovine serum (FBS) (Life Technologies), 100 μg/ml streptomycin and 100 U/ml penicillin (Fisher Scientific) at 37°C in a humidified 5% CO_2_ incubator.

### Uptake of PM2.5

HTR-8 cells at seeding density 0.3 × 10^6^ per well in a 6-well plate were exposed to a single dose of 500 ng/ml of PM2.5 for 24–48 h. After washing with phosphate-buffered saline (PBS), the cells were trypsinized (0.25% trypsin) and pelleted by centrifugation. The cell pellet was fixed for 2 h at RT in fixative (1.5% paraformaldehyde and 1.5% glutaraldehyde in 0.1 M Sörensen buffer pH 7.2), washed once and then stored overnight at 4°C in the Sörensen buffer. The fixed samples were thereafter prepared for ultrathin sectioning and subjected to TEM as reviewed in Carlemalm (33).

### Generation of Reactive Oxygen Species (ROS) and Cytotoxicity

CellTox Green® cytotoxicity assay (Promega) was employed to evaluate cytotoxicity and used according to the manufacturer's protocol. In brief, 1 × 10^4^ cells/well were plated overnight in a 96-well plate. Cells were treated with PM2.5 (1,000, 5,000 and 10,000 ng/ml) for 48 h. The CellTox green dye was diluted 1/500 in CM and applied to the cells. After 15 min of incubation at RT, fluorescence was measured at 485/535 nm using a VICTOR^3^ 1420 Multilabel Counter (Perkin-Elmer) plate reader. ROS was measured by ROS-Glo H_2_O_2_ Assay (Promega) according to the manufacturer's protocol. In short: 1 × 10^4^ cells/well were plated in white, clear-bottom 96-well tissue culture plates, incubated over night to adhere and subsequently exposed to PM2.5 (5,000 and 10,000 ng/ml) for 48 h. The H_2_O_2_ substrate solution (25 μM) was added to each well and incubated for 6 h at 37°C in a CO_2_ incubator. With this, the H_2_O_2_ substrate reacts directly with H_2_O_2_ in the cells and generates a luciferin precursor. Thereafter, ROS-Glo Detection Solution was added and incubated for 20 min at 25°C to generate a luminescence signal. Luminescence was measured using a VICTOR^3^ 1420 Multilabel Counter (Perkin-Elmer) plate reader.

### Protein Secretion

Based on the results obtained from the CellTox Green® cytotoxicity assay, we decided to analyze the level of protein secretion from cells exposed to the following PM2.5 concentrations: 5,000 and 10,000 ng/ml. Cells were seeded at a seeding density of 0.3 × 10^6^ per well in a 6-well plate. After 48 h of exposure, the culture supernatants were collected and used to detect the level of human chorionic gonadotropin (hCG), progesterone and IL-6. Human IL-6 was analyzed using a human IL-6-specific ELISA (Invitrogen) according to manufacturer's protocol. The hCG and progesterone analyzes were performed at the Clinical Biochemistry Laboratory at Lund Hospital, Sweden.

### Mitochondrial Quantification and Function

Cells subjected to different PM exposures (50–10,000 ng/ml) were analyzed for oxygen consumption using an Oroboros O2k-FluoRespirometer (Oroboros Instruments, Innsbruck, Austria) as previously described (34). The cells were loaded into the chamber at 0.5 × 10^6^ cells/ml in complete CM (0.89 oxygen solubility factor was used), with a total cell count of one million cells per chamber, and allowed to stabilize on routine respiration using endogenous substrates. Oligomycin (1 μg/ml) was added to inhibit the ATP-synthase, and the uncoupler protonophore carbonyl cyanide 4-(trifluoromethoxy) phenylhydrazone (FCCP) was then titrated to induce maximum non-coupled respiration. The experiment was terminated by adding rotenone (2 μM) and antimycin (1 μg/ml) to measure non-mitochondrial respiration, a value that was subsequently used to adjust all data for non-mitochondrial cellular oxygen consumption. Citrate Synthase (CS) activity was used as a marker of mitochondrial content as previously described (35). Samples were sonicated and loaded into a 96-well plate in assay buffer with the addition of 300 μM acetyl CoA and 100 μM 5.5-dithiobis-(2-nitrobenzoic acid). In a spectrophotometric plate reader (Bio-Rad Model 680 Microplate Reader; Bio-Rad Laboratories, Hercules, CA, USA) set to 412 nm on a kinetic program with a 1.5-min duration and 10 s intervals, the absorbance of the baseline reaction was measured. This value was deducted from the final reading for each well. Following this, 500 μM of oxaloacetate was added to each well and absorbance was measured. Kinetic plots were individually assessed for a linear change in absorbance and the CS-activity was calculated. Mitochondrial respiratory data was normalized to CS-activity.

Necrosis specific protease, Tripeptidyl peptidase II, was measured by Mitochondrial ToxGlo Assay (Promega) through fluorogenic peptide substrate bis-AAF-R110 according to manufacturer's protocol (36). In brief, cells were seeded at a density of 1 × 10^4^ cells/well in a 96-well plate; after 2 or 48 h of PM2.5 exposure (1,000, 5,000, and 10,000 ng/ml), the cytotoxicity reagent was added to each well, mixed by orbital shaking for 1 min and then incubated at 37°C for 30 min. The fluorescent signal was subsequently measured at 485/535 nm using a VICTOR^3^ 1420 Multilabel Counter (Perkin-Elmer) plate reader.

### PAH Analysis in Cells

Cells were seeded at a seeding density of 0.3 × 10^6^ per well in a 6-well plate and exposed to 10,000 ng/ml of PM2.5 for 48 h. After exposure, supernatants were collected and cells were harvested. Cell pellets were lysed with 500 μl of cell lysis buffer and sonicated for 30 min. Five hundred microliters of water (MilliQ) and 40 ng of internal standards were added to the lysed cells as well as to the supernatants (37). Samples were extracted twice with 2 ml of dichloromethane, and organic extracts were combined and evaporated to near dryness. To each sample, 40 ng of recover standard and 100 μl hexane were added, after which the samples were transferred to HPLC glass vials (Agilent). Analysis of PAHs was performed on a GC-MS 7890 (Agilent) as reported earlier (37).

### Statistical Analysis

Data obtained through the experiments are reported as mean ± SD. Statistical significance between groups was determined by one-way analysis of variance followed by the Tukey's test using OriginPro 2017 software (OriginLab Corporation, Northampton, MA, USA). Statistical significance was defined as *P* < 0.05.

## Results

### Particle Characteristics and Meteorological Data

**Figure 1:**
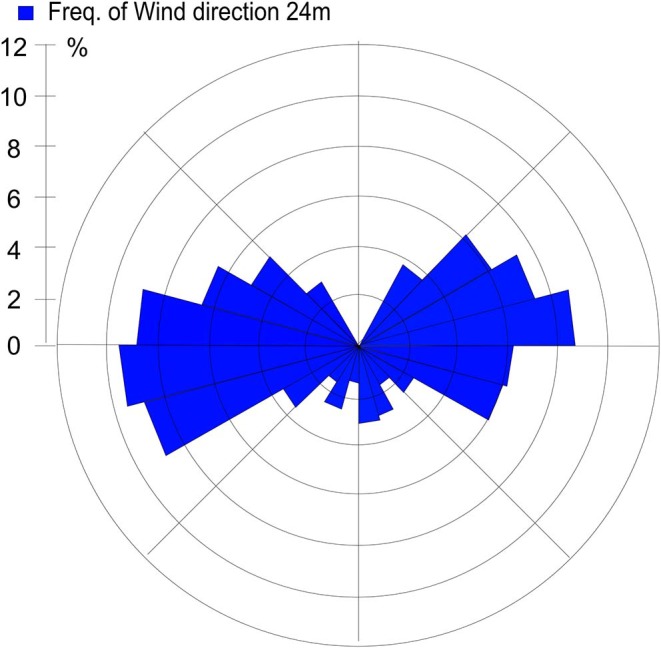
Wind direction frequency from Heleneholmsmasten in Malmö site during the collection period.

**Figure 2:**
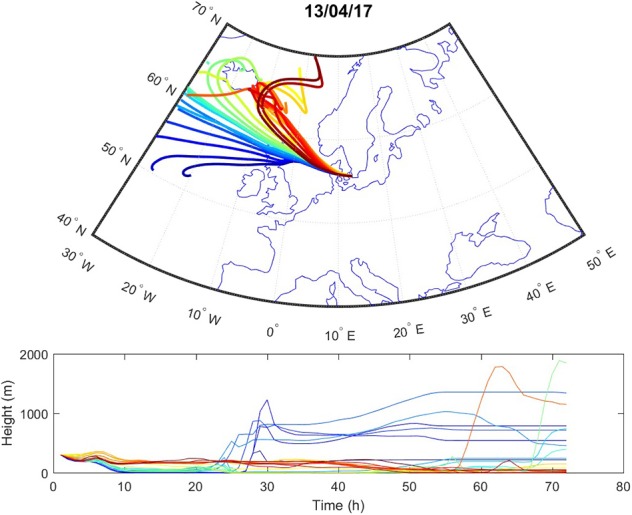
Typical example of air mass origins in Malmö during western winds, trajectories generated using the Hysplit software. The lines show hourly trajectories 72 h backward in time during 1 day.

**Figure 3:**
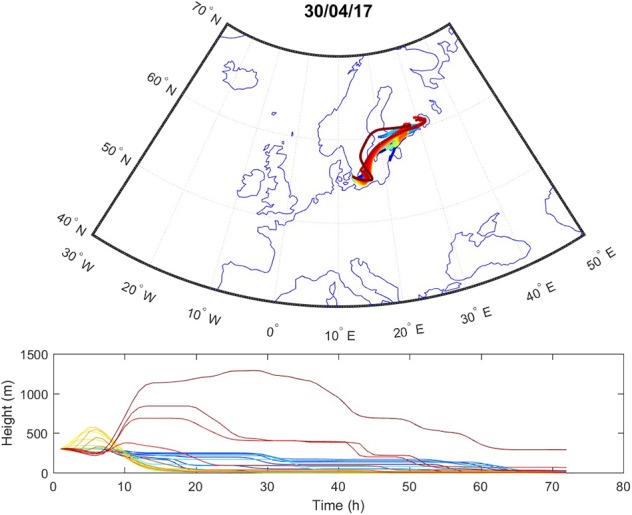
Typical example of air mass origins in Malmö during eastern winds, trajectories generated using the Hysplit software. The lines show hourly trajectories 72 h backward in time during 1 day.

Sources contributing to the collected PM2.5 were assessed in relation to wind direction (Figure 1). The winds during the collection period were predominantly western (48%) or eastern (50%), with only 2% calm winds/air (defined as winds <1 m/s). During these calm periods, all PM can be assumed to be locally generated. When western winds prevail, the major contributive source is Copenhagen, Denmark (Figure 2); on the other hand, air masses typically pass through southern Finland and the Baltic states before reaching Malmö, Sweden during eastern winds (Figure 3). The characteristics of the collected PM2.5 during different wind directions are displayed in Supplementary Table 1. As the table shows, the levels of PM2.5, as well as soot, are higher during calm air, indicating that the majority of all collected PM is locally generated, and is diluted to some extent when wind speeds reach more than 1 m/s. Levels of PAHs and metals in the collected particles can be found in Supplementary Table 2.

### Pregnancy-Related Hormones and Inflammatory Response

**Figure 4:**
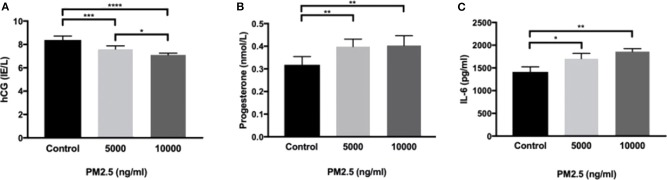
PM2.5 decreases hCG production and increases IL-6 and progesterone production. HTR-8 cells were exposed to PM2.5 for 48 h and subsequently measured for **(A)** hCG, data is presented as mean ± SD; *n* = 6, and *P < 0.05, ***P < 0.001, ****P < 0.0001, **(B)** progesterone, data is presented as mean ± SD; *n* = 6, and **P < 0.01, and **(C)** IL-6, data is presented as mean ± SD; *n* = 3, and *P < 0.05, **P < 0.01 secretion in the CM.

HTR-8 cells exposed to PM2.5 for 48 h showed a significant drop in secreted hCG at 5,000 ng/ml (*P* < 0.001) and 10,000 ng/ml (*P* < 0.0001; Figure 4A), as well as a dose-dependent decrease between 5,000 and 10,000 ng/ml (*P* < 0.05). In addition, there was a significant increase (*P* < 0.01) in progesterone at 5,000 and 10,000 ng/ml (Figure 4B). The PM2.5 exposure also resulted in a significant increase in secretion of IL-6 at 5,000 ng/ml (*P* < 0.05) and 10,000 ng/ml (*P* < 0.01; Figure 4C).

### Cell Viability and H_2_O_2_ Production Affected by PM2.5 Exposure

**Figure 5:**
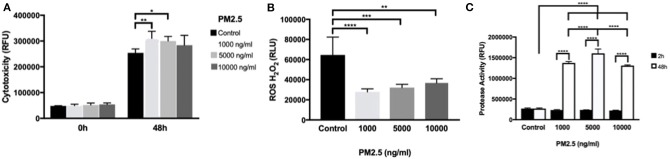
PM2.5 induces cytotoxicity, alters ROS production, and increases protease activity in trophoblast cells. HTR-8 cells were exposed to PM2.5 for 48 h and subsequently analyzed for **(A)** cytotoxicity, data is presented as mean ± SD; *n* = 5 in each experimental condition. *P < 0.05, **P < 0.01 **(B)** ROS production, data is presented as mean ± SD; *n* = 5 in each experimental condition. **P < 0.01, ***P < 0.001, ****P < 0.0001 **(C)** Protease activity, Data is presented as mean ± SD; *n* = 3 in each experimental condition. ***P < 0.001, ****P < 0.0001.

Using the CellTox™ Green Cytotoxicity assay to detect changes in membrane integrity, PM2.5-exposed HTR-8 cells displayed significant cytotoxicity at 1,000 and 5,000 ng/ml at 48 h exposure but not at 10,000 ng/ml compared to controls. At baseline measurements (0 h), there was no difference between the groups (Figure 5A). In the HTR-8 cells, the H_2_O_2_ levels were measured after 48 h of PM2.5 exposure and showed a significant alteration of H_2_O_2_ in all PM2.5-exposed groups (Figure 5B).

To further investigate cell viability, dead cell protease activity was measured at 2 and 48 h of PM2.5 exposure. Protease activity significantly increased (*P* < 0.0001) with cell culture duration, and there was a significant difference in protease activity in all concentrations of PM2.5 compared to controls in additions to significant differences between PM2.5 doses, with peak measurements at 5,000 ng/ml (Figure 5C).

### PM2.5 Uptake: Organelle Localization and Morphological Changes

**Figure 6:**
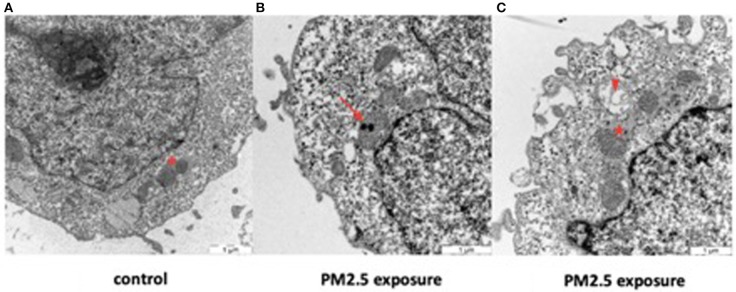
Effect of PM2.5 on mitochondrial morphology in HTR-8 cells. Cells were exposed to a single dose of 500 ng/ml of PM2.5 for 24 or 48 h and subsequently observed through TEM. No differences were seen between the 24 and 48 h exposure groups. Unexposed cells **(A)** showed mitochondria with normal organelle morphology and intact inner and outer membranes (asterisk). In contrast, exposed cells **(B,C)** had internalized PM2.5 particles within the inner membrane of the mitochondria (arrow) and displayed mitochondrial vacuolization (arrowhead), dilated ER structures with large pools of ER lumen (star), and various degrees of mitochondrial membrane disruption. Scale bar = 1 μm.

Transmission electron microscopy of control HTR-8 cells revealed dark and homogenous cytoplasm and mitochondria with intact inner and outer membranes and well-ordered organelle morphology (Figure 6A, Supplementary Figure 3). In comparison, PM2.5-exposed cells displayed several structural changes including mitochondrial vacuolization, aggregated cytoskeleton, chromatin condensation, dilated ER structures, and autophagosomes. The cytoplasm of exposed cells was also lighter in color compared to control cells and contained numerous vacuoles. Finally, the uptake of PM2.5 particles was visualized within the inner mitochondrial membranes of exposed cells (Figures 6B,C, Supplementary Figures 2, 3). Cells were analyzed after 24 and 48 h following exposure, but no differences were noted related to exposure duration, with all of the above-mentioned morphological changes seen in both groups.

### Mitochondrial Function

Neither routine (endogenous) respiration, oligomycin-induced LEAK respiration, maximum non-coupled respiration induced by the protonophore FCCP, nor the control ratio (oligomycin-induced LEAK respiration/maximum non-coupled respiration, Supplementary Figure 1) differed significantly between control cells and cells exposed to various concentrations of PM2.5 for 48 h. Data was normalized to cell number and specific mitochondrial content (citrate synthase activity), respectively, and analyzed without significant findings or trends considered to be consistent or relevant.

### PAH Analysis

Exposed cells and supernatants were analyzed for a set of 16 PAHs previously detected in the collected PM2.5 (Supplementary Table 2). The PAHs could only be detected in cell lysates and not in the supernatants. While PAHs levels were close to the detection limit in cell lysates of untreated cells, PAH concentrations ranged from 7 to 35 ng per sample in cells treated with 10,000 ng PM2.5 (Table 1). Specifically, levels were highest for benzo(b)fluoranthene (35 ng per 10,000 ng PM2.5), pyrene (30 ng per 10,000 ng PM_2.5_) and fluoranthene (27 ng per 10,000 ng PM2.5), followed by phenanthrene, benzo(k)fluoranthene, benzo(a)pyrene, dibenzo(a,h)anthracene, benzo(g,h,i)perylene (13–15 ng per 10,000 ng PM2.5), and anthracene (7 ng per 10,000 ng PM2.5; Table 1). The high concentrations of benzo(b)fluoranthene, pyrene, and fluoranthene at the cellular level are in concordance with the results from the initial PAH analysis of the collected PM2.5 particles (Supplementary Table 2).

**Table 1:** PAH in PM2.5 exposed cells.

**PAH**	**Control cells**	**PM2.5 10,000 ng/ml**
Phenanthrene	2.3	13
Anthracene	1.4	7
Flouranthene	3.8	27
Pyrene	3.2	30
Benzo(b)flouranthene	3.8	35
Benzo(k)flouranthene	–	13
Benzo(a)pyrene	2.5	15
Dibenzo(a,h)anthracene	–	14
Benzo(g,h,i)perylene	1.0	13

## Discussion

Ambient air pollution in the form of particles is the single largest environmental health threat of our time (13). Studies on the effects of air pollution on human health pertaining to biological pathways and biological effects have mainly focused on the respiratory organs; therefore, more studies on adverse health effects during pregnancy are highly warranted (38). In fact, a growing body of evidence suggests an association between air pollution and birth- and pregnancy outcomes (17, 39). Even though all components of air pollution are harmful to human health, particulate matter in particular can consist of, and carry, a broad range of toxic substances that, depending on particle size and particle solubility, can penetrate the respiratory tract and gain access to the blood stream (40, 41). These such PM compositions are particularly dangerous for pregnancies.

Pregnancy encompasses a period in which susceptibility to exposure-related alterations is heightened, initiating adverse processes that can have life-long implications for both mother and child. Throughout pregnancy, the placenta serves as a gatekeeper between mother and fetus. Indeed, the fetus is indirectly in contact with the same environmental stressors as the mother through the maternal blood circulation. This makes the placenta a useful, albeit temporary, organ for studies on accumulated pregnancy exposures, such as ambient air pollution.

Indeed, a recent study reports evidence of inhaled PM being translocated from the lungs to the placenta (42). Although the placenta's actual exposure to PM2.5 is unknown, we attempted to study doses relevant to human exposure. Levels up to 5,000 ng/ml, for example, could correspond to 25 μg/m^3^, which is the WHO target for short term exposure (24 h), and 10,000 ng/ml might correspond to levels in a more polluted city with PM2.5 concentrations of 50 μg/m^3^. Previous studies have estimated the physiological range to be similar to the doses used in this study (43).

PM exposure in general has been linked to inhibition of phagocytosis, stimulation of inflammatory response, and increases in levels of oxidative stress (44). Even specific toxic compounds connected to PM, such as PAHs, are known to be carcinogenic and genotoxic and have also been shown to disrupt endocrine functions (45, 46). In addition, several studies have demonstrated that PAH exposure during pregnancy is associated with adverse pregnancy outcomes, such as small for gestational age (SGA) or IUGR (47). In this study, the molecular effects of PM2.5 on first trimester trophoblast cells were evaluated by studying mechanisms related to cytotoxicity, cellular disruption, mitochondrial function, inflammatory response, and hormone production. We have previously conducted a study of the effects of PM2.5 on trophoblast cells showing a reduction in cellular growth, endoplasmic reticulum stress, and altered protein expression (43). In this study, we confirm our previous results as well as broaden the scope to include additional organelle systems.

In relation to inflammatory response, Interleukin 6 (IL-6) a pro-inflammatory cytokine and, an anti-inflammatory myokine was examined. It has effects on both cells from the immune system and other cell types, such as hepatocytes (48). Interleukin-6 is mainly secreted by monocytes but is also produced by trophoblast cells *in vivo* (49) as well as *in vitro* by the HTR-8 trophoblast cell line (50). Interleukin 6 binds to the IL-6 receptor, expressed either on the surface of cells or in a soluble form released by several cell types. This pro-inflammatory cytokine, together with IL-8 and TNF-a, have been suggested as potential inflammatory markers for PE (51), increased plasma levels has been associated with adverse pregnancy outcomes such as PE (52, 53). Our results demonstrate that PM2.5 exposure elevates the production of IL-6 in the HTR-8 cell line. Previous studies have shown that IL-6 stimulation of trophoblast cells induces an increase in hCG production (54). The results from our study diverge from these findings: cells treated with PM2.5 responded with an increased production of IL-6 and a decreased production of hCG. This might be due to possible PM2.5 interference with the steroidogenesis in the trophoblast cells or other upstream mechanisms. The increased production of IL-6 indicate that the trophoblast cells were in a heightened inflammatory state; moreover, they lacked the necessary increase in hCG, which is pivotal to maintaining viable and healthy placental development. In fact, pregnancy-associated diseases such as PE, IUGR and preterm births, all have defect placentation in combination with aggravated inflammatory responses as common denominators (55, 56). One could, therefore, speculate that the imbalance between the increased inflammation caused by IL-6 and the absence of hCG increase could be part of the explanation as to why air pollution has been linked to these pregnancy complications (57) as well as PE and IUGR (17, 58). During the initial phase of pregnancy progesterone is produced by corpus luteum, but by the end of the first trimester the progesterone production is in full taken over by the trophoblast cells. In this study we used a first trimester cell line, and it is somewhat surprising that we observe an increase in progesterone. The progesterone and hCG production in trophoblasts are interconnected by a range of mediators (59). The fact that hCG and progesterone jointly drive the trophoblasts to form a syncytium in the very early stages in pregnancy, and that disturbances have been observed in PE and IUGR, our increase in progesterone it is an interesting finding (60, 61). The levels of progesterone were significantly elevated, although close to the detection limit of the assay.

Experimental studies have established an association between exposure to PM2.5 containing high levels of metals or PAH and increased ROS production in the exposed cells (62). Correspondingly, our previous and current results illustrate that trophoblast cells absorb PAHs and, thereafter, exhibit an altered production of ROS (43). Considering that ROS is a major driver of the progression of PE, through systemic inflammation and vascular endothelial damage, these results are unexpected and allude to the complexity of PM2.5's impact on biological systems.

The mitochondria are central to upholding the oxidation-reduction balance, also called redox balance in the cell, being both a target for, and producer of ROS (63). Enhanced levels of ROS tilt the cellular redox balance, which in turn trigger inflammation and eventually apoptosis (64, 65). Exposure to PM2.5 has been shown to target mitochondria specifically and induce mitochondrial ROS production in human lung cells (66). The smaller particles, ultrafine particles (UFPs), have been shown to localize to mitochondria of endothelial cells, where they induce major structural damage (67). Combustion-related particles have also been shown to cause mitochondrial structural damage in lung cells (68, 69). Studies on placentas exposed to higher levels of air pollution show placental mitochondrial DNA methylation (70) and decreased placental mitochondrial DNA content (71). Such mitochondrial dysfunction has been proposed to be one of the mechanisms contributing to the dysfunctional placenta in PE (72).

Our results confirm that PM2.5 specifically targets the mitochondria in the trophoblast cells, visualized by the localization of the particles to this organelle. The structural damage of the mitochondria observed by TEM is a direct indicator of mitochondrial dysfunction. Potential explanations for this dysfunction could include PM2.5's physical presence localized to the mitochondria, its interference with redox balance and molecular processes in the mitochondria and/or its chemical PAH content. Indeed, the chemical content of the particles may add to the cellular toxicity observed. Our findings showed that the same PAHs were found in the PM2.5 particles were also discovered in the, suggesting that PAH may disorb, or leak, from the particles in a biological matrix, thus being able to cause cell damage. Despite the limited sample size of the PAH analysis on cell lysates, these results clearly indicate that there is an uptake of PAH into the cells.

No dose-dependent effect of exposure to PM2.5 on mitochondrial respiration was found in this study. We did, however, notice a somewhat surprising decline in H_2_O_2_ production in the exposed cells. One could speculate that this might be due to the increased cytotoxicity present in the PM2.5 exposed cells, which would effectively leave fewer viable cells able to produce H_2_O_2_. Cells exposed to a single dose of PM2.5 showed a significant decrease in cell count after 7 days (Supplementary Figure 4). Cytotoxicity at the highest doses is not statistically significant compared to the control, therefore it would be difficult to draw any conclusions. One could speculate if reduced proliferation as a result of exposure to high doses of PM2.5, could explain this finding. Similar speculations of reduced proliferation can be made regarding the protease activity being lower in the higher fraction of PM2.5. In the PM2.5 exposed groups there was a trend of increasing ROS-production with increasing dosage. However, due to the small sample size, statistical significance was not upheld. Further studies are needed to determine the effects of PM2.5 on ROS-production. In addition to the local effects of the PM2.5 in the mitochondria, pro-inflammatory factors such as IL-6 have been suggested to induce mitochondrial damage (73). The results from this study not only confirms that the particles were internalized by the cells, as observed by TEM imaging (Figure 6), but also showed that PAHs could be released from the particles inside the cells, thus having a possible role in inducing the cellular toxicity observed.

The HTR-8 trophoblast cell line does not form a syncytia, a potential drawback of this study, however the HTR-8 trophoblast cell line is a first trimester cell line and hence displaying age typical characteristics for the period of gestation when the pathological changes to the placenta associated with PE is thought to occur. Future avenues to explore could entail syncytia forming trophoblast, placental organoid cultures and/or the dual-placenta-perfusion model.

To understand the correlation between PM2.5 exposure and PE, as well as other adverse pregnancy outcomes, it is essential to gain further knowledge of the mechanisms through which PM2.5 interferes with placental function during pregnancy. Air pollution has already been linked to placental transcriptome changes (74), placental DNA methylation (75) and a reduced placenta weight (76). Further, women exposed to high levels of air pollution from cooking with solid fuels have shown chronic placental hypoxia (77) and fetal thrombotic vasculopathy (78). Adding to this body of knowledge, our data suggest that exposure to PM2.5 *in vitro* has the potential to induce cellular toxicity as well as hormone dysregulation, oxidative damage, inflammatory response, and interference with mitochondria in first trimester placental cells (HTR-8 cells). Future studies will have to elucidate if theses findings are translatable to *in vivo* conditions.

## Public Health Impact

The effects of air pollution on pregnancy and birth outcomes are not negligible. One study estimated that 11% of term low birth weight cases would be avoided if the PM2.5 concentration was reduced by 5 μg/m^3^ (58). Another suggests that 11% of all PE cases were attributable to exhaust emissions in Malmö, Sweden (79). Indeed, the PM2.5 used in our present study was collected in real-life conditions in Malmö, Sweden in order to mimic the actual exposure pregnant women face in an urban setting. Our findings on the subsequent effects on human trophoblasts cells are suggestive of underlying placental mechanisms potentially contributing to the adverse effects air pollution exposure exerts during pregnancy.

## Data Availability Statement

The datasets generated for this study are available on request to the corresponding author.

## Author Contributions

ÅN, LE, EM, CI, BS, AK, TL, and SH conceived and designed the experiments. ÅN, LE, AK, EÅ, CI, and MS performed the experiments. ÅN, LE, EM, CI, MS, EE, JE, BS, TL, AK, and SH analyzed the data. ÅN, LE, EM, and SH wrote the paper. ÅN, LE, CI, MS, EÅ, JE, AK, BS, TL, EE, EM, and SH revised the manuscript. ÅN, LE, CI, MS, EÅ, JE, AK, BS, TL, EE, EM, and SH approved the final version of the manuscript.

### Conflict of Interest

SH holds patents for the diagnosis and treatment of preeclampsia. SH is co-founder of the companies Preelumina Diagnostics AB and A1M pharma AB. The remaining authors declare that the research was conducted in the absence of any commercial or financial relationships that could be construed as a potential conflict of interest.

## Abbreviations

PE: preeclampsia
PM2.5: particulate matter of size <2.5 μm
PAHs: polycyclic aromatic hydrocarbons
PM: particulate matter
IL-6: Interleukin 6
ROS: Reactive Oxygen Species
TEM: transmission electron microscopy
RT: room temperature
hCG: human chorionic gonadotropin
SD: standard deviation
PBS: phosphate-buffered saline
CS: citrate synthase
IUGR: intra uterine growth restriction
NCEP: Centre of Environmental Predictions
GDAS: global data assimilation system.
