# Supplementary material for: Urban PM2.5 Induces Cellular Toxicity, Hormone Dysregulation, Oxidative Damage, Inflammation, and Mitochondrial Interference in the HRT8 Trophoblast Cell Line
Source: Front Endocrinol (Lausanne). 2020 Mar 12;11:75. doi: 10.3389/fendo.2020.00075 (PMC7080655; doi:10.3389/fendo.2020.00075)
Supplement: Supplementary file 1 [file Data_Sheet_1.docx]

Supplementary Table 1. Levels of various components in Malmö ambient air during the collection period.

| Component (Average values) | Eastern winds | Western winds | Calm |
| --- | --- | --- | --- |
| PM 2.5 (µg/m^3^) | 6.2 | 6.0 | 7.3 |
| CO (mg/m^3^) | 0.15 | 0.17 | 0.26 |
| CO_2_ (ppm) | 415 | 415 | 428 |
| O_3_ (µg/m^3^) | 69.6 | 67.8 | 43.6 |
| NO (µg/m^3^) | 7.9 | 12.3 | 10.3 |
| NO_2_ (µg/m^3^) | 18.3 | 20.9 | 33.8 |
| Soot (BC measured at 880 nm) (ng/m^3^) | 397 | 350 | 555 |
| Local biomass burning (% of soot) | 23 | 24 | 20 |

Supplementary Table 2. PAH and metal concentrations in collected PM2.5.

| **PAH** | **Concentration (ng/mg)** | **Metal** | **Concentration (ng/mg)** |
| --- | --- | --- | --- |
| *naphtalene* | <0.039 | Aluminium (Al) | 330 |
| 2-methylnaphtalene | <0.052 | Vanadium (V) | 36 |
| 1-methylnaphtalene | <0.031 | Chromium (Cr) | 4.8 |
| biphenyl | <0.0082 | Manganese (Mn) | 34 |
| 2,3-dimethylnaphtalene | <0.012 | Iron (Fe) | 890 |
| *acenaphthylene* | 0.044 | Cobalt (Co) | 1.8 |
| *acenaphtnene* | <0.051 | Nickel (Ni) | 14 |
| 2,3,5-trimethylnaphtalene | <0.0062 | Copper (Cu) | 100 |
| *fluorene* | 0.075 | Zink (Zn) | 240 |
| 1-methylfluorene | 0.038 | Arsenic (As) | 5.3 |
| *phenanthrene* | 0.96 | Cadmium (Cd) | 0.49 |
| *anthracene* | 0.075 | Barium (Ba) | 17 |
| 2-methylphenanthrene | 0.34 | Thallium (Tl) | 0.061 |
| 3-methylphenanthrene | 0.26 | Lead (Pb) | 14 |
| 1-methylphenanthrene | 0.25 |  |  |
| 1-methylanthracene | 0.24 |  |  |
| 2-phenhylnaphtalene | 0.29 |  |  |
| *fluoranthene* | 2.7 |  |  |
| *pyrene* | 3.0 |  |  |
| 1-methylfluoranthene | <0.00034 |  |  |
| 1-methylpyrene | 0.17 |  |  |
| retene | 0.60 |  |  |
| *benzo(a)anthracene* | 1.3 |  |  |
| *chrysene* | 3.9 |  |  |
| 2-methylchrysene | 0.31 |  |  |
| *benzo(b)fluoranthene* | 4.2 |  |  |
| *benzo(k)fluoranthene* | 3.1 |  |  |
| *benzo(a)pyrene* | 0.77 |  |  |
| perylene | 0.15 |  |  |
| *indeno(1,2,3-c,d)pyrene* | 2.1 |  |  |
| *dibenzo(a,h)anthracene* | 0.39 |  |  |
| *benzo(g,h,i)perylene* | 2.6 |  |  |
|  |  |  |  |
| Sum PAHs | 28 |  |  |
| Sum 16 US EPA PAHs | 25 |  |  |
| Sum acrylated PAHs | 2.6 |  |  |

Supplementary Figure 1.

Mitochondrial respiration in trophoblasts exposed to various concentrations on PM2.5 particles for 48 hours. Control ratio (oligomycin-induced LEAK respiration / maximum inducible non-coupled mitochondrial respiration). Data from n 4 – 6 (n = 2 for conc. 1000). No significant difference between groups using non-parametric Kruskal Wallis test of variation. Data is shown as mean ± SEM.

Supplementary Figure 2.


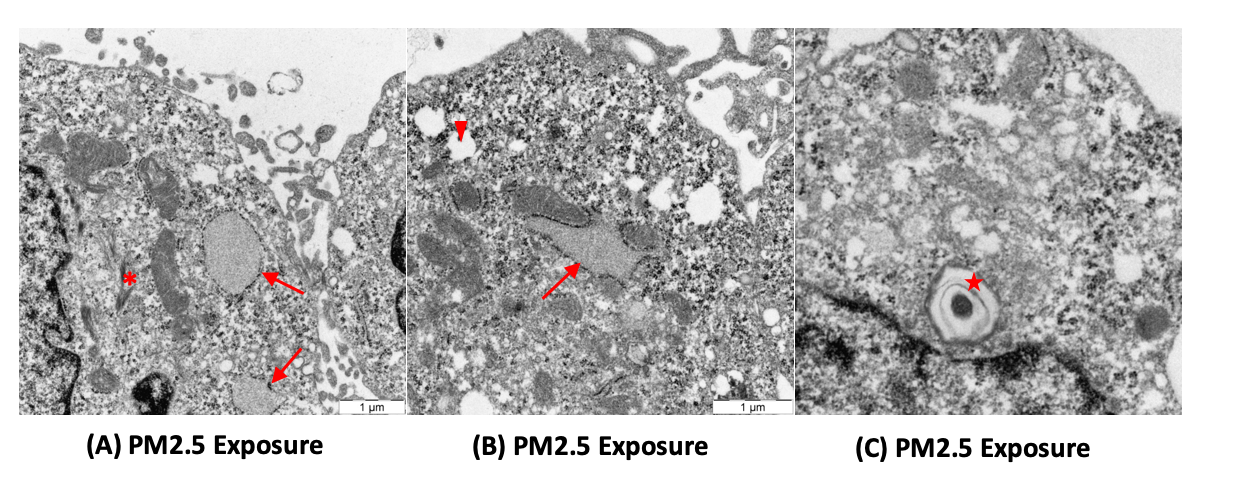


**Effect of PM2.5 on mitochondrial morphology in HTR-8 cells**. Cells exposed to a single dose of 500 ng/ml of PM2.5 and thereafter observed by TEM displayed aggregated cytoskeleton (asterisk), dilated ER structures (arrows), mitochondrial vacuolization (arrowhead), and autophagosomes (star). Scale bar = 1 um.

Supplementary Figure 3.


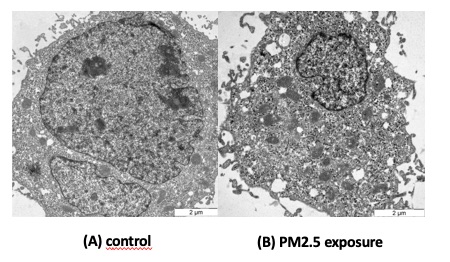


**Effect of PM2.5 on mitochondrial morphology in HTR-8 cells**. Cells exposed to a single dose of 500 ng/ml of PM2.5 and thereafter observed by TEM.

Supplementary Figure 4.


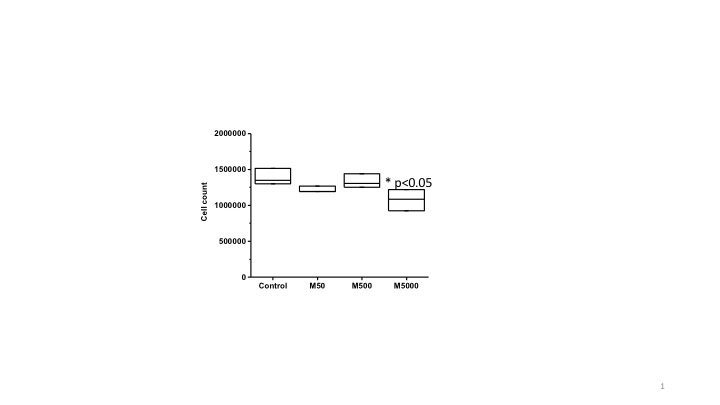


**Cell count of HTR-8 cells exposed to PM2.5.** Cells exposed to various concentrations (50-5000 ng/ml) PM2.5 particles in a single dose at 24 h and cells were harvested and counted after 7 days showed a significant decrease in cell count (p=0.05).
